# Adverse childhood experiences across male offender subgroups in Bavaria

**DOI:** 10.1192/j.eurpsy.2026.10947

**Published:** 2026-07-31

**Authors:** S. Schobel, C. Blunck, K. Schiltz, M. Wertz

**Affiliations:** Department of Forensic Psychiatry, University Hospital, Ludwig Maximilians University Munich, Munich, Germany

## Abstract

**Introduction:**

Adverse childhood experiences (ACEs), such as abuse and neglect, constitute significant risk factors for engagement in criminal behavior. Previous research has highlighted high rates of ACEs in incarcerated and forensic psychiatric populations, while less is known about potential differences in the prevalence and types of ACEs across offender subgroups.

**Objectives:**

To examine the prevalence and types of adverse childhood experiences in a male offender population and to explore potential differences across offender subgroups.

**Methods:**

The study included N = 311 male offenders, comprising 183 prison inmates and 128 forensic psychiatric patients in Bavaria. Among the participants, 33.5% had committed violent offenses, 16.1% sexual offenses, 23.8% drug-related offenses, and the remaining 26.6% other types of offenses. Adverse childhood experiences were assessed using the self-report Childhood Trauma Questionnaire (CTQ). Differences in ACE prevalence and types across offender subgroups were analyzed.

**Results:**

Regarding adverse childhood experiences, 10.9 % of participants indicated moderate–severe and 19.6 % severe–extreme emotional abuse. Physical abuse was reported by 10.3 % (moderate–severe) and 26.7 % (severe–extreme), while sexual abuse was indicated by 6.5 % and 12.1 %, respectively. Emotional neglect affected 14.8 % (moderate–severe) and 21.5 % (severe–extreme), and physical neglect 19 % and 23.5 %, respectively. Analyses across offender subgroups in regards to type of offense revealed a significant effect only for sexual abuse. Post-hoc comparisons indicated that individuals convicted of sexual offenses reported significantly higher levels of sexual abuse compared to individuals convicted of drug-related offenses. No significant differences were observed regarding institutional placement. A significant effect of prior convictions was found for physical abuse and physical neglect, with higher scores observed in participants with a history of prior convictions. Additionally, small negative correlations were observed between age at onset of delinquent behavior and both physical abuse and physical neglect.

**Conclusions:**

The high prevalence of adverse childhood experiences among male offenders highlights the critical role of early trauma in pathways to criminal behavior. Differences across offense types and links with criminal history suggest that specific forms of adversity may influence offending patterns and recidivism. Limitations include the cross-sectional design and reliance on self-report measures, which may be subject to recall or reporting bias. Future research should explore longitudinal effects and intervention outcomes.

**Disclosure of Interest:**

None Declared

